# Impact of poor muscle strength on clinical and service outcomes of older people during both acute illness and after recovery

**DOI:** 10.1186/s12877-017-0512-6

**Published:** 2017-06-07

**Authors:** Salah Gariballa, Awad Alessa

**Affiliations:** 1 Department of Internal Medicine, Faculty of Medicine and Health Sciences, United Arab Emirates University, PO Box 17666, Al-Ain, United Arab Emirates; 2 University of Sheffield, Sheffield, UK

## Abstract

**Background:**

Although Low muscle strength is an important predictor of functional decline in older people, however information on its impact on clinical and service outcomes in acute care settings is still lacking. The aim of this study is to measure the impact of low muscle strength on clinical and service outcomes in older adults during both acute illness and recovery.

**Methods:**

Randomly selected 432 hospitalised older patients had their clinical characteristics and nutritional status assessed within 72 h of admission, at 6 weeks and at 6 months. Low muscle strength-hand grip was defined using the European Working Group criteria. Health outcome measures including nutritional status, length of hospital stay, disability, discharge destination, readmission and mortality were also measured.

**Results:**

Among the 432 patients recruited, 308 (79%) had low muscle strength at baseline. Corresponding figures at 6 weeks and at 6 months were 140 (73%) and 158 (75%). Patients with poor muscle strength were significantly older, increasingly disabled, malnourished and stayed longer in hospital compared with those with normal muscle strength. A significantly higher number of patients with normal muscle strength discharged home independently compared with those with poor muscle strength (*p* < 0.05). One-year death rate was lower in patients with normal muscle strength 5(6%), compared with those with poor muscle strength 52(15%), however, results were not statistically significant after adjusting for other poor prognostic indicators [adjusted hazard ratio 0.74 (95% CI: 0.14–3.87), *p* = 0.722].

**Conclusion:**

Poor muscle strength in older people is associated with poor clinical service outcomes during both acute illness and recovery.

## Background

The number of people aged 65 years and over is growing rapidly worldwide and projected to increase further in the future. Ageing in man is associated with physiological and pathological changes many of which have impact on treatment and prevention of disease and maintenance of good health. For example, both muscle strength and mass deteriorate with ageing and are known to be associated with disability in later life [1–3]. Loss of muscle strength over time is known to be greater than loss of muscle mass [4–6]. Furthermore longitudinal studies have revealed that decline in muscle strength in older people far exceeds the observed changes in muscle mass and that treatments that maintain or increase muscle mass may not necessarily decrease or prevent muscle weakness in later life [6, 7]. Many cross-sectional and prospective studies have revealed that muscle strength is an indicator of functional decline in community free living older people [8–10]. Furthermore recurrent ill health is more common in older people and that inflammatory response during acute illness leads to a state of negative nitrogen balance resulting in significant loss of muscle mass. The loss of muscle mass if significant may lead to poor clinical outcome [3]. Although poor muscle strength has emerged as an important predictor of frailty data on hospitalized patients are lacking. Knowledge of underlying causes and health impact of poor muscle strength is expected to help guide management and therefore reduce adverse outcomes [7, 11–13]. The aim of this study is to measure the impact of poor muscle strength on important health outcomes of older patients during both acute illness and recovery.

## Methods

### Subjects

A randomly selected sample of 432 acutely ill hospitalized older patients with complete data was included [12]. All acutely ill older people admitted to Barnsley District General Hospital 7 days a week were considered for the study. Barnsley District General Hospital serves a total population of 234,000. It has 650 beds; the medical unit has 250 beds for acute medical admissions. Subjects were first identified through the computerised databases of all patients in hospital. When first admitted all patients have an individualised computerised plan created. This allowed all patients to be screened for suitability including those admitted over the week end. The medical notes of those identified from the database were examined and eligible patients approached. Common admission diagnoses of study population include coronary heart disease, chest, urine and blood infections, chronic obstructive lung disease, heart failure, falls, stroke, syncope, diabetes and arthritis**.** Patients included in the study were those aged ≥65 years and medically stable. Unstable patients with severe medical or psychiatric illness and those living in institution were excluded from the study. The Barnsley Research Ethics Committee approved the study and written consent was obtained from all recruited patients.

### Clinical and nutritional assessment

All patients had clinical and nutritional baseline assessment within 72 h of admission in hospital and at 6 weeks and 6 months either in hospital or at home. Recruited subjects had the following data collected: demographic and medical data, current diagnosis, and history of chronic illnesses, smoking, alcohol and drug intake, nutritional status, disability, length of hospital stay, discharge destination, readmission and mortality. The Barthel score was used to measure disability. The Barthel score is a reliable score for assessing disability and scores 10 functions on a scale 0 (fully dependent) to 20 (independent) [13]. The scores were recorded after direct assessment of the patient, discussion with the nurses in charge of the patient or from the records documented by the multi-disciplinary staff involved in the assessment and treatment of that individual patient. Patients were followed up until death or discharge and at up to 12 months.

Anthropometric, hematological and biochemical measurements were used for assessment of nutritional status [12]. All anthropometric measurements were performed using standard methods validated prior to the commencement of the study. Mid-arm circumference (MAC) was measured by a flexible tape. Triceps skin folds (TSF) was measured using Happened Skin fold calipers accurate to 0.2 mm (Practical Metrology Sussex UK). Routine tests including haemoglobin, albumin and transferrin were performed by the local pathology laboratory. Severity of illness (inflammation) was assessed using C-reactive protein (CRP) concentration. CRP was measured by a modified latex-enhanced immuno-turbidimetric assay (normal range **≤** 10 mg/L). The inter-assay coefficient of variation (C.V.) was 3.9%.

### Muscle strength-hand grip [2, 3]

A dynamometer (Practical Metrology, Sussex, and UK) was used for measuring handgrip strength. Three measurements were taken from the dominant hand unless this was unusable (recent stroke weakness). Low muscle strength was defined using the cut-off points of the European Working Group on Sarcopenia in Older people. Low muscle strength = hand grip less than 30 kg and 20 kg in men and women respectively.

Using,

### Statistical analyses

SPSS software, version 22 (SPSS Inc., Chicago) used for statistical analyses. Independent student-*t* test or the nonparametric Mann-u-Whitney was used depending on data distribution to test between group differences with a *p*-value of <0.05 regarded as statistically significant. A proportional hazards model was used to examine 1-year mortality between patients with low handgrip-muscle strength and those with normal strength after adjusting for age, gender, disability, comorbidity, body mass index (BMI), and serum albumin. The Kaplan-Meier (K-M) survival curve used to assess the risks of death.

## Results

**Fig. 1:**
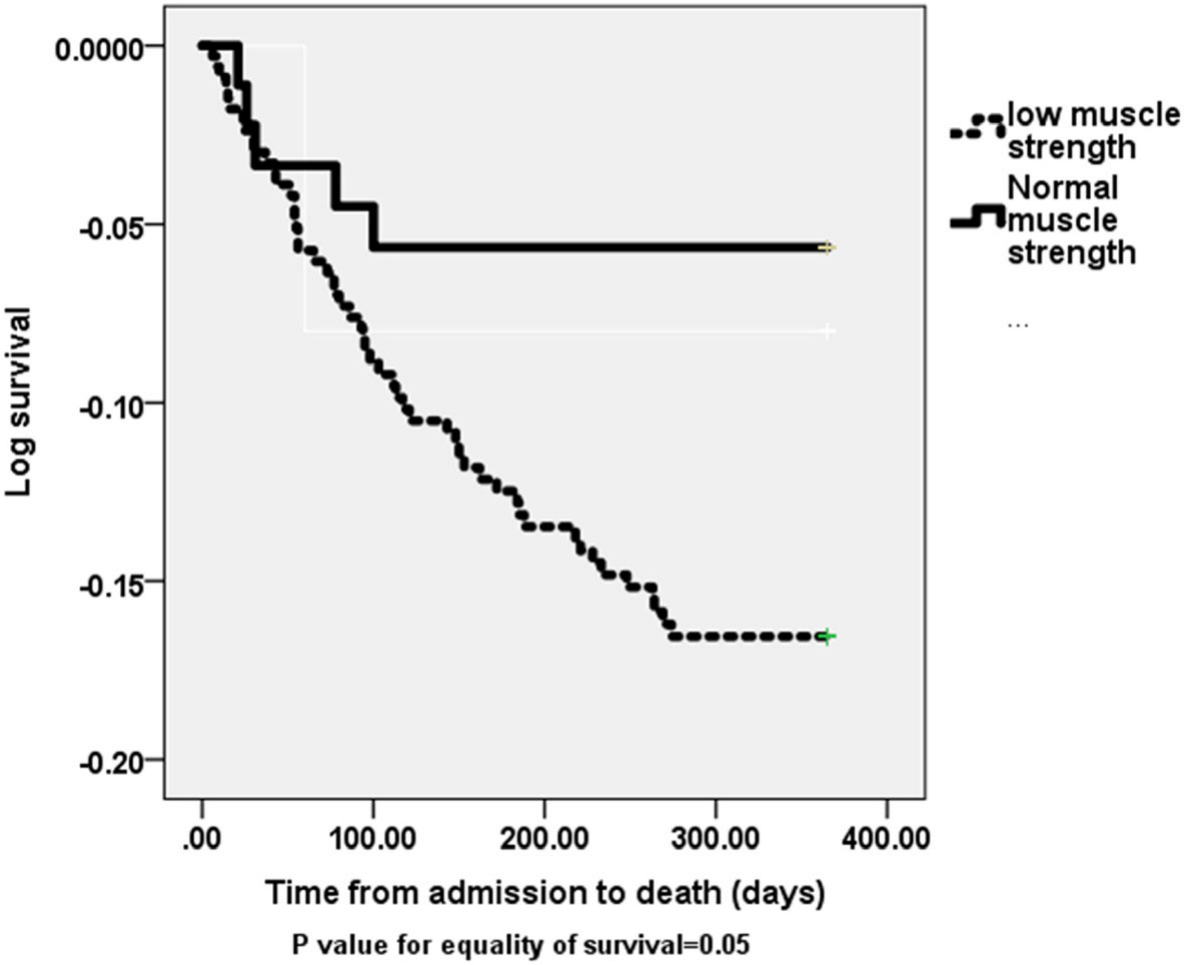
Kaplan-Meier Log survival curve for 1 year mortality for study patients with low baseline handgrip-muscle strength compared with those with normal handgrip-muscle strength

**Fig. 2:**
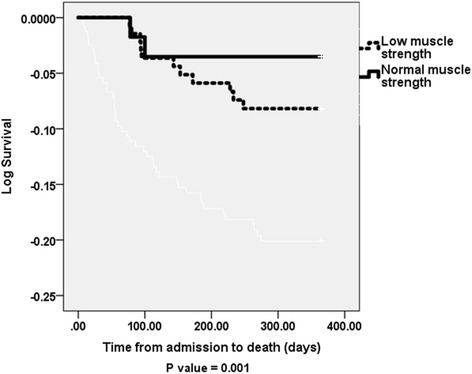
Kaplan-Meier Log survival curve for 1 year mortality for study patients with low 6-week handgrip-muscle strength compared with those with normal handgrip-muscle strength

All 432 acutely ill older patients admitted to hospital and followed up for period of 12 months were included in this analysis. Among the 432 patients recruited 308 (79%) had low muscle strength at baseline. Figures at 6 weeks and at 6 months were 140 (73%) and 158 (75%). Exclusions were due to early death or inability to provide outcome data at follow up visits. Baseline characteristics of study population are shown in Table 1. Patients with poor muscle strength were significantly older with increased disability and poor nutritional status compared with those with normal muscle strength (Table 1). Table 2 shows clinical and service outcome measures. Patients with poor muscle strength had significantly longer length of stay in hospital (LOS) compared to patients with normal muscle strength [LOS 10.3 (7) versus 8.3 (6) days respectively, *p* = 0.027]. A higher number of subjects with normal muscle strength discharged home independently compared with those with poor muscle strength, *p* > 0.05 (Table 2). One-year death rate was significantly lower in patients with normal muscle strength measured on admission or at 6 weeks compared with those with poor muscle strength, *p*-value = <0.05, (Table 2). Stratified analysis by gender revealed men with low muscle strength had significantly longer LOS, increased disability and mortality compared with men with normal muscle strength (*p* < 0.05). Result for women were only significant for those needing assistance at 6 months (Table 3). Using Cox regression analysis adjusted difference in mortality between patients with low muscle strength and those with normal strength measured on admission and at 6 weeks were however not statistically significant [adjusted hazard ratios were 0.53 (95% CI: 0.21–1.3), *p* = 0.166, and 0.74 (95% CI: 0.14–3.87, *p* = 0.722) respectively] (Tables 4 and 5, Figs. 1 and 2).

**Table 1:** Baseline characteristics of subjects with low handgrip-muscle strength compared with those with normal handgrip-muscle strength, mean (SD), unless stated otherwise

Variable		Low muscle strength (*n* = 341)	Normal muscle strength (*n* = 91)	*P* value
Age (years)		77.5 (6)	77 (6)	0.000
Gender, female, *n* (%)		190 (56)	15 (17)	0.000
Smoking, *n* (%)	Never smoked	111 (33)	24 (26)	0.900
	Ex-smoker	160 (47)	56 (62)	
	Current smoker	70 (20)	11 (12)	
Chronic disease/patient, (n)		2	1.6	0.078
Drugs/patient, (n)		3.6	1.6	0.052
Barthel Score		15.3 (4.8)	16.1 (4.6)	0.000
Body mass index		24.7 (4)	26.8 (3)	0.000
Triceps skinfold thickness		15.7 (7)	15.3 (6)	0.585
C-reactive protein mg/L		53 (73)	49 (72)	0.687
Haemoglobin g/dl		12.6 (2)	13.4 (2)	0.001
Albumin g/L		37.5 (5)	39.2 (4)	0.002
Transferrin g/L		2.17 (0.53)	2.11 (0.41)	0.379

*n* (%) = number of patients (percentage)

**Table 2:** Clinical outcome measures for study patients with low baseline handgrip-muscle strength compared with those with normal handgrip-muscle strength, mean (SD), unless stated otherwise

Variable	Low muscle strength (*n* = 341)	Normal muscle strength (*n* = 91)	*P* value
Length of hospital stay (days)	10.2 (7)	8.3 (6)	0.027
Disability at 6 weeks	18.1 (2.5)	19.6 (1)	0.000
Disability at 6 months	18.3 (3)	19.6 (1)	0.000
Discharge to own home, *n* (%)	141 (41)	63 (69)	0.000
Needing assistance at 6 weeks *n* (%)	52 (37)	9 (16)	0.005
Needing assistance at 6 months *n* (%)	100 (44)	14 (19)	0.000
6-month readmission, *n* (%)	121 (36)	28 (31)	0.401
6-month mortality, *n* (%)	45 (13)	5 (6)	0.042
12-month mortality, *n* (%)	52 (15)	5 (6)	0.015

*n* (%) = number of patients (percentage)

**Table 3:** Clinical outcome measures for study patients with low baseline handgrip-muscle strength compared with those with normal handgrip-muscle strength stratified by gender, mean (SD) unless stated otherwise

Variable	Female		Male	
	low strength *n* = 190	Normal strength *n* = 15	low strength *n* = 151	Normal strength *n* = 76
Length of hospital stay (days)	9.4 (6)	7.5 (3)	11.2 (9)	8.4 (7)^*^
Disability at 6 weeks	18.2 (3)	19.4 (1)	18.3 (2)	19.3 (2)^*^
Disability at 6 months	18 (3)	19.2 (1)	18.3 (3)	19.4 (1)
Discharge to own home, *n* (%)	80 (42)	10 (67)	61 (40)	53 (70)^*^
Needing assistance at 6 weeks, *n* (%)	27 (14)	1 (7)	25 (17)	8 (11)^*^
Needing assistance at 6 months, *n* (%)	56 (30)	1 (7) ^*^	44 (29)	13 (17)
6-month readmission, *n* (%)	65 (34)	3 (20)	56 (37)	25 (32)
6-month mortality, *n* (%)	16 (8)	0	29 (19)	5 (7)
12-month mortality, *n* (%)	20 (11)	0	32 (21)	5 (7)^*^

^*^ *P* < 0.05

*n* (%) = number of patients (percentage)

**Table 4:** The Cox’s proportional hazard analysis of the influence of admission handgrip-muscle strength and other prognostic variables on 1-year mortality

Variable	Regression coefficient	Standard error	*P* value	Hazard ratio for unit change	95.0% CI	
					Lower	Upper
Age (years)	.052	.027	.056	1.053	.999	1.111
Gender (male/female)	−1.142	.324	.000	.319	.169	.602
Barthel Score (0–20)	−.050	.032	.122	.951	.893	1.013
Chronic disease	.164	.099	.097	1.179	.971	1.431
Smoking (Never, Ex, Current)	.474	.225	.035	1.607	1.033	2.500
Body mass index	−.033	.038	.393	.968	.898	1.043
C-reactive protein (mg/L)	−.001	.002	.676	.999	.994	1.004
Serum albumin (g/L)	−.110	.038	.004	.895	.831	.965
Handgrip strength (kg)	−.638	.461	.166	.528	.214	1.304

**Table 5:** The Cox’s proportional hazard analysis of the influence of handgrip-muscle strength at 6 weeks and other prognostic variables on 1-year mortality

Variable	Regression coefficient	Standard error	*P* value	Hazard ratio for unit change	95.0% CI	
					Lower	Upper
Age (years)	.149	.062	.017	1.160	1.027	1.310
Gender (male/female)	−.865	.767	.259	.421	.094	1.893
Barthel Score (0–20)	−.177	.097	.069	.838	.692	1.014
Chronic disease	.258	.207	.213	1.294	.863	1.942
Smoking (Never, Ex, Current)	.513	.519	.324	1.669	.603	4.620
Body mass index	−.144	.103	.163	.866	.708	1.060
C-reactive protein (mg/L)	−.006	.010	.545	.994	.973	1.014
Serum albumin (g/L)	−.099	.085	.242	.906	.767	1.069
Handgrip strength (kg)	−.301	.845	.722	.740	.141	3.875

## Discussion

In this study, we showed that older patients with poor muscle strength particularly men had poor health outcomes during both acute illness and recovery.

Well recognized determinants of poor muscle strength in older patients during both acute illness and after recovery include age, gender, chronic diseases and disability and tissue inflammation [14]. After adjustment for most of these poor prognostic indicators it was still possible to identify a potentially independent effect of poor muscle strength on patient’s health outcomes. We have also excluded all unstable severely ill patients or those living in an institution from the study. Excluded patients were more likely to have low muscle strength and this might have underestimated the prevalence of poor health outcomes in our study population. Although mortality was higher in patients with poor muscle strength measured during both acute illness and recovery, this relationship was not statistically significant after adjusting for poor prognostic indicators. This result highlights the complex relationship between poor muscle function/nutrition, increasing age, disability and underlying co morbidity in clinical practice. Nevertheless muscle strength is now an important marker of sarcopenia and been proposed as a useful single predictor of generalized frailty [14, 15]. Although many studies have identified an association between poor muscle strength, increasing frailty and morbidity in free living older people in the community, very few studies have addressed its’ impact on service and health outcomes during both acute illness and recovery. For example, a cross-sectional study from the UK assessed grip strength in 47 patients in rehabilitation and 100 nursing home residents reported lower grip strength than for people living at home [14]. Similar studies from Europe and North America reported lower grip strength for subjects in rehabilitation and care home settings [16, 17].

When results are stratified by gender our findings are broadly in agreement with a recent study from Mexico which showed that male patients with low handgrip-muscle strength at admission to acute care facility had an increased risk of functional decline at discharge [18].

Although, a relationship between health parameters and changes in body composition in older people has been reported, a common underlying pathophysiological mechanism linking changes in muscle and fat mass with muscle strength and functional decline is not well defined. A cross-section study on 672 women aged 65 years and older reported an independent association between oxidative protein damage and low grip-strength suggesting an involvement of increased oxidative stress in loss of muscle strength in older people [19]. Another recent cross-sectional survey reported an association between C-reactive protein a marker of inflammation and low hand-grip strength in men and women aged 65–74 years [20]. The association between low muscle strength on admission and poor outcome is partly explained by age, gender and underlying co morbidity including low serum albumin and acute inflammation as a result of acute illness. Acute and some chronic illnesses in older people for example, lead to tissue inflammation and release of inflammatory markers. These markers lead to many symptoms such as fever, loss of appetite and alteration in body metabolism. These changes consequently lead to decrease food intake and also reduced body weight and muscle function therefore contributing to development of low muscle mass and increased disability in older people. Nevertheless grip strength represent the newest approach for evaluating nutritional status however similar to other nutritional status measurement parameters is affected by age-related changes and disease [21]. Finding a plausible underlying mechanism linking muscle function with poor health is clearly an area for future research.

A number of approaches for improvement of poor muscle function in older people have been explored [22]. Nutrition supplement and exercise in particular deserve special attention in acutely ill older patients with poor muscle function. Firstly, because acutely ill older patients with poor muscle strength are more likely to have decrease in physical activity and poor nutritional intake prior to the acute illness. Their nutritional intake and status is likely to deteriorate further as a result of the acute illness and during the period of hospitalization and rehabilitation [20]. Secondly following acute illness older people become physically inactive and many will not regain their premorbid physical activity levels for some time after recovery from the illness. This is clinically relevant because physical activity benefits most risk factors of ageing patients including muscle function. Dietary protein may also have a role in the maintenance of muscle mass and function in older people [23, 24]. Adequate amount of high quality protein intake which provides essential amino acids in combination with physical activity may improve muscle mass, function and therefore delay the onset of sarcopenia [22]. More research is needed however on the effect of increased physical activity and high quality protein intake in the treatment of poor muscle function following acute illness.

### Study strength and limitations

We have no data on pre admission and long term post discharge dietary intake. Another limitation is the number of exclusion at follow up visits and difficulties related to measurements of nutritional indices in ageing patients. The purpose of assessing validity of anthropometric measurements, the longitudinal design of the study and adjustments for poor prognostic clinical indicators during the analysis was to overcome some of these weaknesses.

## Conclusions

In conclusion this study shows that poor muscle strength is associated with poor health outcomes in hospitalized older patients during both acute illness and recovery. Research combining clinical trials with basic molecular investigations is needed to fully understand the role of increase physical activity combined with adequate intake of high quality dietary protein particularly following acute illness on muscle strength and mass in ageing patients. Meanwhile patients with poor muscle strength may benefit from targeted nutritional assessment and support.

## Abbreviations

C.V: Coefficient of variation
CRP: C-reactive protein
MAC: Mid-arm circumference
TSF: Triceps skin folds

## References

[1] Sayer AA (2010). Sarcopenia: a research agenda has been set, but recognition in clinical practice is lagging behind. BMJ.

[2] Cruz-Jentoft AJ, Baeyens JP, Bauer JM, Boirie Y, Cederholm T, Landi F (2010). European working group on sarcopenia in older people sarcopenia: European consensus on definition and diagnosis: report of the European working group on sarcopenia in older people. Age Ageing.

[3] Landi F, Liperoti R, Russo A, Giovannini S, Tosato M, Capoluongo E, Bernabei R, Onder G. Sarcopenia as a risk factor for falls in elderly individuals: Results from the ilSIRENTE study. Clin Nutr. 2012. doi:10.1016/j.clnu.2012.02.007. 10.1016/j.clnu.2012.02.007 22414775

[4] Frontera WR, Reid KF, Phillips EM, Krivickas LS, Hughes VA, Roubenoff R (2008). Muscle fiber size and function in elderly humans. J Appl Physiol.

[5] Goodpaster BH, Park SW, Harris TB, Kritchevsky SB, Nevitt M, Schwartz AV (2006). The loss of skeletal muscle strength, mass, and quality in older adults: the health, aging and body composition study. J Gerontol A Biol Sci Med Sci.

[6] Hughes VA, Frontera WR, Wood M, Evans WJ, Dallal GE, Roubenoff R (2001). Longitudinal muscle strength changes in older adults: influence of muscle mass, physical activity, and health. J Gerontol A Biol Sci Med Sci.

[7] Cesari M, Fielding RA, Pahor M, Goodpaster B, Hellerstein M (2012). Biomarkers of sarcopenia in clinical trials-recommendations from the international working group on sarcopenia. J Cachex Sarcopenia Muscle.

[8] Rantanen T, Guralnik JM, Foley D, Masaki K, Leveille S, Curb JD, et al. Midlife hand grip strength as a predictor of old age disability. JAMA. 1999;281:558–60. 10.1001/jama.281.6.558 10022113

[9] Hicks GE, Shardell M, Alley DE, Miller RR, Bandinelli S, Guralnik J, et al. Absolute strength and loss of strength as predictors of mobility decline in older adults: the InCHIANTI study. J Gerontol A Biol Sci Med Sci. 2012;67:66–73. 10.1093/gerona/glr055 PMC3260485 21546582

[10] Stenholm S, Rantanen T, Heliovaara M, Koskinen S (2008). The mediating role of C-reactive protein and handgrip strength between obesity and walking limitation. J Am Geriatr Soc.

[11] Fielding RA, Vellas B, Evans WJ, Bhasin S, Morley JE, Newman AB (2011). Sarcopenia: an undiagnosed condition in older adults. Current consensus definition: prevalence, etiology, and consequences. International working group on sarcopenia. J Am Med Dir Assoc.

[12] Gariballa S, Alessa A (2013). Sarcopenia: prevalence and prognostic significance in hospitalized patients. Clin Nutr.

[13] Collin C, Wade DT, Davies S, Horne V (1988). The Barthel ADL index: a reliability study. Int Disabil Stud.

[14] Roberts HC, Syddall HE, Sparkes J, Ritchie J, Butchart J, Kerr A (2014). Grip strength and its determinants among older people in different healthcare settings. Age Ageing.

[15] Syddall H, Cooper C, Martin F, Briggs R, Aihie SA (2003). Is grip strength a useful single marker of frailty?. Age Ageing.

[16] McAniff CM, Bohannon RW (2002). Validity of grip strength dynamometry in acute rehabilitation. J Phys Ther Sci.

[17] Guerra RS, Amaral TF (2009). Comparison of hand dynamometers in elderly people. J Nutr Health Ageing.

[18] García-Peña C, García-Fabela LC, Gutiérrez-Robledo LM, García-González JJ, Arango-Lopera VE (2013). Pérez-Zepeda MU handgrip strength predicts functional decline at discharge in hospitalized male elderly: a hospital cohort study. PLoS One.

[19] Howard C, Ferrucci L, Sun K, Fried LP, Walston J, Varadhan R, et al. Oxidative protein damage is associated with poor grip strength among older women living in the community. J Appl Physiol (1985). 2007;103(1):17–20. 10.1152/japplphysiol.00133.2007 PMC2646087 17379753

[20] Sousa AC, Zunzunegui MV, Li A, Phillips SP, Guralnik JM, Guerra RO (2016). Association between C-reactive protein and physical performance in older populations: results from the international mobility in aging study (IMIAS). Age Ageing.

[21] Gariballa SE (2001). Malnutrition in hospitalised elderly patients: when does it matter?. Clin Nutr.

[22] Kung T, Springer J, Doehner W, Anker SD, von HS. (2010). Novel treatment approaches to cachexia and sarcopenia: highlights from the 5th cachexia conference. Expert Opin Investig Drugs.

[23] Paddon-Jones D, Short KR, Campbell WW, Volpi E, Wolfe RR (2008). Role of dietary protein in the sarcopenia of aging. Am J Clin Nutr.

[24] Paddon-Jones D, Campbell WW, Jacques PF, Kritchevsky SB, Moore LL, Rodriguez NR, et al. Protein and healthy aging. Am J Clin Nutr. 2015;101:1339S. 10.3945/ajcn.114.084061 25926511

